# Influenza Vaccination During Pregnancy: A Narrative Review on Maternal and Neonatal Outcomes Associated with Seasonal Influenza Infection

**DOI:** 10.3390/vaccines14070593

**Published:** 2026-07-03

**Authors:** María Morales-Suárez-Varela, Isabel Peraita-Costa, Agustín Llopis-Morales, Agustín Llopis-González

**Affiliations:** 1 Research Group in Social and Nutritional Epidemiology, Pharmacoepidemiology and Public Health, Department of Preventive Medicine and Public Health, Food Sciences, Toxicology and Forensic Medicine, Faculty of Pharmacy and Food Sciences, Universitat de València, Av. Vicent Andrés Estelles 22, 46100 València, Spain; isabel.peraita@uv.es (I.P.-C.); allomo@alumni.uv.es (A.L.-M.); agustin.llopis@uv.es (A.L.-G.); 2 Biomedical Research Center in Epidemiology and Public Health Network (CIBERESP)–CIBER de Epidemiologia y Salud Pública (CIBERESP), Carlos III Health Institute, Av. Monforte de Lemos 3-5 Pabellón 11 Planta 0, 28029 Madrid, Spain

**Keywords:** influenza, pregnancy, maternal, vaccination congenital malformations, neonatal outcomes, influenza vaccine, preterm birth, maternal immunization

## Abstract

Seasonal influenza remains an important public health concern worldwide, and pregnant women represent a particularly vulnerable population due to physiological and immunological changes associated with gestation. Influenza infection during pregnancy has been associated with adverse maternal, fetal and neonatal outcomes. This narrative review summarizes current evidence regarding maternal influenza infection and influenza vaccination during pregnancy. A structured literature search was conducted using PubMed, Embase and Cochrane Library databases. Studies published between 2020 and 2025 addressing maternal influenza infection, pregnancy outcomes and influenza vaccination were reviewed. Current evidence suggests that maternal influenza infection is associated with increased risks of spontaneous abortion, preterm birth, hospitalization and congenital malformations, especially neural tube defects and congenital heart defects when infection occurs during the first trimester. In contrast, evidence regarding long-term neurodevelopmental outcomes remains inconsistent. Influenza vaccination during pregnancy demonstrates moderate-to-high effectiveness in preventing maternal and neonatal influenza infection and shows a favorable safety profile. Available evidence also suggests that neuraminidase inhibitors, particularly oseltamivir, can be used safely during pregnancy without increasing the risk of congenital malformations or adverse neonatal outcomes. Influenza vaccination during pregnancy should continue to be promoted as a safe and effective public health strategy to protect both mothers and infants.

## 1. Introduction

Seasonal influenza remains one of the most important respiratory infectious diseases worldwide and continues to represent a major public health concern because of its substantial morbidity, mortality and economic burden. According to the World Health Organization (WHO), annual influenza epidemics are associated with millions of severe cases and hundreds of thousands of respiratory deaths every year [1]. Although influenza infection affects all age groups, certain populations are particularly vulnerable to severe disease, including older adults, immunocompromised individuals and pregnant women.

Pregnancy is characterized by profound physiological, cardiovascular and immunological changes that may increase susceptibility to respiratory viral infections and severe influenza-related complications [2]. Adaptations in the maternal immune system are essential to maintain fetal tolerance during gestation; However, these immunological modifications may also impair the maternal response against viral pathogens. Furthermore, respiratory and cardiovascular adaptations occurring during pregnancy, including increased oxygen consumption, diaphragm elevation and reduced lung capacity, may contribute to worse clinical outcomes in pregnant women with influenza infection [3].

Historical evidence from previous influenza pandemics, particularly the 1918 H1N1 pandemic and the 2009 influenza A/H1N1 pandemic, demonstrated disproportionately higher morbidity and mortality rates among pregnant women compared with the general population [4]. During the 2009 pandemic, pregnant women presented increased risks of hospitalization, intensive care unit admission and severe respiratory complications [5]. Consequently, pregnancy is currently recognized as an independent risk factor for severe influenza infection.

Beyond maternal morbidity, influenza infection during pregnancy has also raised concerns regarding fetal and neonatal health outcomes. Several studies have suggested associations between maternal influenza infection and adverse pregnancy outcomes, including spontaneous abortion, preterm birth, fetal growth restriction and congenital malformations [6,7]. In particular, first-trimester influenza infection has been associated with increased risks of neural tube defects and congenital heart defects, potentially mediated by maternal hyperthermia, placental dysfunction and systemic inflammatory responses [8].

Additionally, maternal immune activation during pregnancy has been proposed as a possible mechanism linking prenatal viral infections with altered fetal neurodevelopment [9]. Experimental and epidemiological studies have explored whether maternal influenza infection could contribute to long-term neurodevelopmental or neuropsychiatric disorders in offspring, including autism spectrum disorder, schizophrenia and attention-deficit/hyperactivity disorder. However, available evidence remains inconsistent, and causality has not been definitively established [10].

Influenza vaccination during pregnancy is considered the most effective strategy for preventing influenza-related maternal and neonatal morbidity [11]. International organizations including the WHO and the Centers for Disease Control and Prevention (CDC) recommend seasonal influenza vaccination for pregnant women during any trimester of pregnancy [12]. Maternal vaccination not only protects pregnant women against severe influenza infection but also provides passive immunity to infants during the first months of life through transplacental antibody transfer and breast milk antibodies [13]. This neonatal protection is especially relevant because infants younger than six months are not eligible for influenza vaccination and remain particularly vulnerable to severe influenza infection.

Beyond the protective effects observed in mothers and infants, influenza vaccination during pregnancy represents an important public health intervention because it contributes to reducing healthcare utilization, influenza-related hospitalizations, and the burden on healthcare systems during seasonal epidemics. However, vaccine effectiveness may vary between influenza seasons due to antigenic drift and the continuous evolution of circulating influenza strains. This variability highlights the importance of annual vaccine reformulation and ongoing surveillance programs to optimize vaccine strain selection and maintain adequate levels of protection.

Despite strong recommendations supporting maternal influenza vaccination, vaccination coverage among pregnant women remains suboptimal in many countries [14]. Vaccine hesitancy, concerns regarding vaccine safety, misinformation and limited healthcare professional recommendation continue to represent major barriers to vaccine uptake during pregnancy [15]. Consequently, improving awareness regarding the safety and effectiveness of maternal influenza immunization remains a priority public health objective.

The aim of this narrative review is to provide an updated and clinically oriented synthesis of current evidence regarding maternal influenza infection, pregnancy outcomes, and the effectiveness and safety of influenza vaccination during pregnancy.

## 2. Narrative Review Methodology

### 2.1. Study Design

This study was conducted as a narrative review using a structured literature search strategy. The review was designed to summarize and critically discuss recent evidence regarding seasonal influenza infection during pregnancy, maternal and neonatal outcomes, and the role of influenza vaccination as a preventive strategy rather than to perform a formal systematic review or quantitative meta-analysis. To improve transparency in reporting and reproducibility, selected elements of the PRISMA 2020 statement [16] were considered when describing the search strategy and study selection process. However, this review should be interpreted as a narrative review and not as a fully PRISMA-compliant systematic review.

### 2.2. Literature Search Strategy

A structured search of the scientific literature was conducted using the electronic databases PubMed/MEDLINE, Embase and Cochrane Library. The search process was performed between October 2025 and January 2026.

The search strategy included combinations of Medical Subject Headings (MeSH) terms and free-text keywords related to influenza infection, pregnancy and vaccination. The main search terms included:

“influenza” OR “flu” AND “pregnancy” OR “pregnant women” AND “influenza vaccination” OR “maternal immunization” AND “congenital malformations” OR “preterm birth” OR “neonatal outcomes”.

Boolean operators (AND/OR) were used to optimize the search strategy and improve sensitivity. Additional manual searches of reference lists from selected articles were also performed to identify potentially relevant studies.

### 2.3. Eligibility Criteria

Studies were considered eligible for inclusion if they met the following criteria:

- Published between January 2020 and December 2025.
- Written in English or Spanish.
- Focused on seasonal influenza infection during pregnancy and/or influenza vaccination in pregnant women.
- Evaluated maternal, fetal, neonatal or vaccination-related outcomes.
- Corresponded to systematic reviews, meta-analyses, randomized clinical trials or high-quality observational studies.

The following publication types were excluded:

- Editorials, comments and conference abstracts.
- Animal-only studies without clinical relevance.
- Studies unrelated to pregnancy or influenza infection.
- Articles lacking sufficient methodological or outcome information.

### 2.4. Study Selection

**Figure 1:**
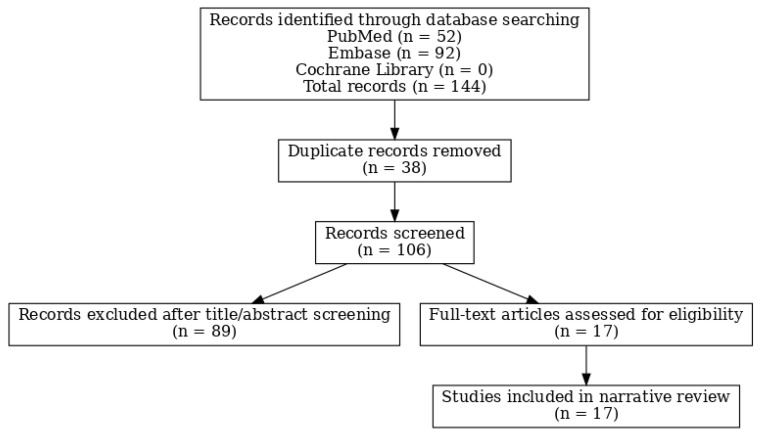
PRISMA flow diagram of study selection.

Titles and abstracts identified through the database search were screened to evaluate relevance according to the predefined inclusion and exclusion criteria (Figure 1). Full-text articles were subsequently reviewed when eligibility could not be established from the abstract alone. The literature screening and study selection process was conducted independently by two reviewers (M.M.S.-V. and I.P.-C.). Titles and abstracts were initially screened for relevance, followed by full-text assessment of potentially eligible articles. Any disagreements regarding study eligibility were resolved through discussion and consensus among the authors. This approach was adopted to improve consistency in study selection and minimize potential selection bias.

The selected studies were grouped according to thematic relevance into the following categories:

1. Maternal and obstetric outcomes associated with influenza infection.
2. Fetal and neonatal outcomes associated with maternal influenza infection.
3. Influenza vaccination during pregnancy.
4. Antiviral treatment during pregnancy.

Particular attention was paid to studies evaluating congenital malformations, neurodevelopmental outcomes, prematurity and vaccine effectiveness because these represent the most clinically relevant and controversial areas in the current literature.

### 2.5. Data Extraction and Synthesis

Relevant information from each selected study was extracted manually. Data extraction was conducted independently by the same two reviewers using a standardized framework. Extracted information included study design, sample characteristics, exposure definitions, outcomes evaluated, principal findings, and reported limitations. The extracted information was subsequently reviewed by all authors to ensure consistency and accuracy of the narrative synthesis. The extracted variables included:

- Author and year of publication.
- Study design.
- Population and sample size.
- Main exposure evaluated.
- Maternal, fetal or neonatal outcomes.
- Vaccine effectiveness and safety outcomes.
- Main conclusions and limitations.

Given the heterogeneity of study designs, populations and outcome measures, a quantitative meta-analysis was not performed. Instead, findings were synthesized narratively to provide a clinically oriented overview of the available evidence.

### 2.6. Risk of Bias and Quality Assessment

Whenever possible, methodological quality and risk of bias were considered according to the design of the included studies. Furthermore, because this study was designed as a narrative review rather than a formal systematic review, a quantitative risk-of-bias assessment was not performed for each included study. However, methodological quality was considered during study selection and interpretation of the evidence. Preference was given to systematic reviews, meta-analyses, randomized clinical trials, and high-quality observational studies because they provide higher levels of scientific evidence. For systematic reviews and meta-analyses, attention was paid to methodological rigor and reporting quality according to principles incorporated in tools such as AMSTAR-2 [17]. For observational studies, aspects commonly evaluated by the Newcastle–Ottawa Scale [18,19], including participant selection, comparability of study groups, and outcome assessment, were considered qualitatively when interpreting study findings. The present review also considered the methodological limitations reported by the original studies, including heterogeneity, residual confounding, retrospective data collection, recall bias and variability in influenza diagnostic methods. Potential overlap among primary studies included in different systematic reviews and meta-analyses was considered during evidence interpretation.

### 2.7. Ethical Considerations

This study was based exclusively on previously published scientific literature and did not involve human participants, patient data collection or experimental interventions. Therefore, ethical approval and informed consent were not required.

## 3. Results

### 3.1. Maternal and Obstetric Outcomes Associated with Influenza Infection

The studies included in this review consistently showed that influenza infection during pregnancy is associated with adverse maternal and obstetric outcomes (Table 1). Wang et al. (2021) [6] reported an increased risk of spontaneous abortion among pregnant women infected with influenza, with an odds ratio (OR) of 1.38. Similarly, Wang et al. (2023) [7] demonstrated a significant association between maternal influenza infection and preterm birth (OR 1.52). These findings suggest that maternal inflammatory responses induced by influenza infection may contribute to early labor and pregnancy loss.

Additional evidence provided by Jiang et al. (2026) [20] reinforced the relationship between influenza infection and pregnancy complications, including miscarriage and prematurity. Their experimental findings proposed ferroptosis, oxidative stress and inflammatory pathways as potential mechanisms underlying fetal injury and placental dysfunction.

**Table 1:** Studies on maternal and obstetric outcomes associated with influenza during pregnancy.

Study	Design	Sample	Outcome Evaluated	Main Findings	Interpretation	Key Limitations
Wang et al., 2021 [6]	Systematic review and meta-analysis	17 studies; 2,351,204 pregnant women	Miscarriage	Increased risk of spontaneous abortion (OR 1.38)	Influenza may contribute to early pregnancy loss	Heterogeneity between included studies and outcome definitions.
Wang et al., 2023 [7]	Meta-analysis	24 studies; 24,760,890 pregnant women with influenza or SARS-CoV-2	Preterm birth	Increased risk of prematurity (OR 1.52)	Maternal infection may trigger inflammatory pathways associated with early labor	High statistical heterogeneity among studies.
Gao et al., 2025 [21]	Systematic review	58 studies; 2,102,294 influenza patients	Hospitalization	Pregnancy increased hospitalization risk (OR 1.88)	Pregnancy identified as risk factor for severe influenza	Heterogeneity between included studies, sparse reporting and lack of standardized definitions.
Jiang et al., 2026 [20]	Experimental study with supporting systematic review and meta-analysis	Experimental animal model; literature review component included	Pregnancy complications	Increased miscarriage and prematurity risk	Ferroptosis and oxidative stress proposed as mechanisms	Findings are based primarily on a chicken embryo model.

OR: odds ratio.

The biological plausibility of the observed associations is supported by emerging evidence suggesting that maternal immune activation, oxidative stress, placental dysfunction, and ferroptosis-related mechanisms may influence fetal development. However, the relative contribution of these pathways remains incompletely understood and requires further investigation.

The magnitude of these associations varied across studies, reflecting differences in study design, influenza diagnostic criteria, and adjustment for confounding variables. However, the consistency of findings across systematic reviews and meta-analyses strengthens the evidence supporting an association between maternal influenza infection and adverse obstetric outcomes. Several authors have suggested that systemic inflammatory responses, placental dysfunction, and altered maternal immune regulation may contribute to these complications. Although causality cannot be definitively established from the available observational evidence, the overall direction of the findings remains remarkably consistent across different populations and healthcare settings.

Pregnancy itself was also identified as an independent risk factor for severe influenza infection. Gao et al. (2025) [21] observed increased odds of hospitalization among pregnant women with influenza (OR 1.88), supporting the concept that gestation represents a state of increased vulnerability to severe respiratory viral disease.

### 3.2. Fetal and Neonatal Outcomes Associated with Maternal Influenza Infection

Studies evaluating fetal and neonatal outcomes showed heterogeneous findings (Table 2).

Fung et al. (2022) [10] found no consistent associations between prenatal influenza exposure and autism spectrum disorder, attention-deficit/hyperactivity disorder or schizophrenia. Likewise, San Martín-González et al. (2023) [9] reported inconsistent evidence regarding psychomotor and neurodevelopmental alterations among exposed offspring. Wiegersma et al. (2025) [22] also failed to identify clear associations between prenatal influenza exposure and dementia risk later in life.

In contrast, studies evaluating congenital malformations demonstrated more consistent results. Mátrai et al. (2022) [8] identified increased risks of neural tube defects, spina bifida, cleft lip/palate and congenital heart defects following first-trimester maternal influenza infection. Similarly, Su et al. (2026) [23] confirmed a significant association between first-trimester influenza infection and congenital heart defects.

Furthermore, Arabzadeh et al. (2024) [24] classified maternal influenza infection as a highly suggestive risk factor for neural tube defects. Collectively, these findings suggest an association between maternal influenza infection during early pregnancy and an increased risk of certain congenital malformations. Although inflammatory, hyperthermic, and placental mechanisms have been proposed, the available evidence remains largely observational and does not establish a definitive causal relationship.

Finally, Rimawi et al. (2022) [25] described possible temporal associations between maternal influenza infection and fetal arrhythmias, although current evidence remains limited and insufficient to establish causality.

An important distinction emerges when comparing congenital and neurodevelopmental outcomes. Evidence regarding congenital malformations, particularly neural tube defects and congenital heart defects following first-trimester exposure, appears relatively consistent across systematic reviews and meta-analyses. In contrast, studies evaluating long-term neurodevelopmental outcomes have produced heterogeneous results. Differences in follow-up duration, diagnostic criteria, outcome definitions, and methods used to ascertain maternal infection may partly explain these discrepancies. Consequently, the current evidence provides stronger support for an association between maternal influenza infection and congenital anomalies than for long-term neurodevelopmental disorders.

**Table 2:** Studies on fetal and neonatal outcomes associated with maternal influenza infection.

Study	Design	Sample	Outcome Evaluated	Main Findings	Interpretation	Key Limitations
Fung et al., 2022 [10]	Systematic review	42 studies	Neuropsychiatric disorders	Evidence regarding schizophrenia, ASD, and other neurodevelopmental disorders was inconsistent and insufficient for causal conclusions.	Evidence remains heterogeneous	High heterogeneity and varied exposure definitions.
Mátrai et al., 2022 [8]	Systematic review and meta-analysis	14 studies	Congenital malformations	First-trimester influenza increased the odds of any non-chromosomal congenital anomaly (OR 1.50), neural tube defects (OR 2.48), cleft lip/palate (OR 2.48), and congenital heart defects (OR 1.63).	First trimester identified as critical exposure period	Predominantly observational evidence; residual confounding cannot be excluded.
Rimawi et al., 2022 [25]	Narrative review and case reports	2 cases and literature review; Pregnant women with influenza	Fetal arrhythmias	Possible temporal association between influenza and fetal arrhythmias	Evidence limited and not conclusive	Limited evidence base.
San Martín-González et al., 2023 [9]	Systematic review	13 studies	Neurodevelopment	Maternal respiratory viral infections, including influenza, may be associated with subtle motor, behavioral, and attentional alterations in offspring.	No definitive causal relationship established	Small number of studies and potential confounding factors.
Arabzadeh et al., 2024 [24]	Umbrella review	Multiple meta-analyses	Neural tube defects	Maternal influenza was associated with increased NTD risk (OR 3.33), although evidence strength was classified as suggestive rather than conclusive.	Reinforces association with congenital anomalies	Based on pooled observational studies with varying quality.
Su et al., 2026 [23]	Meta-analysis	1,732,295 pregnancies (30 studies)	Congenital heart defects	First-trimester maternal infection was associated with increased CHD risk; influenza specifically showed OR 1.50 (95% CI 1.20–1.87).	Supports teratogenic role of influenza-related inflammation	Many studies relied on self-reported infections.
Wiegersma et al., 2025 [22]	Systematic review		Dementia risk	No clear increase in Alzheimer disease or vascular dementia	Long-term effects remain uncertain	Evidence is highly susceptible to confounding and bias.

ASD: autism spectrum disorder; CHD: congenital heart defects; OR: odds ratio.

### 3.3. Influenza Vaccination During Pregnancy

Current evidence strongly supports the effectiveness and safety of maternal influenza vaccination (Table 3).

Omer et al. (2020) [11] reported vaccine effectiveness ranging from 35% to 56% in infants during the first six months of life and approximately 50% effectiveness in mothers during pregnancy and postpartum. These findings highlight the importance of maternal immunization in protecting both mothers and newborns.

Muthiani et al. (2023) [26] found no significant reductions in low birth weight or prematurity associated with vaccination; However, vaccination effectively reduced influenza infection risk without increasing adverse pregnancy outcomes.

Motsoeneng et al. (2024) [27] demonstrated that influenza vaccination induced measurable immunological responses associated with significantly lower infection risk as similarly did Chittajallu et al. (2025) [14]. Finally, Deese et al. (2025) [13] identified increased anti-influenza IgA antibodies in breast milk after maternal vaccination, supporting the role of breastfeeding in passive neonatal immunity.

Beyond the individual studies summarized in Table 3, the overall body of evidence consistently supports influenza vaccination during pregnancy as a safe and effective intervention for both maternal and neonatal protection. Vaccine effectiveness varies across influenza seasons and geographical settings because of differences between vaccine strains and circulating viruses, particularly as a consequence of antigenic drift. However, even during seasons with suboptimal strain matching, vaccination has been associated with reduced risks of laboratory-confirmed influenza infection, influenza-related hospitalization, and severe respiratory complications among pregnant women.

The safety profile of influenza vaccination during pregnancy has been extensively evaluated in randomized clinical trials, observational studies, and systematic reviews. Available evidence does not support an increased risk of miscarriage, congenital malformations, preterm birth, low birth weight, stillbirth, or adverse neonatal outcomes associated with maternal vaccination. These findings have been consistent across different trimesters of administration and across multiple influenza vaccine formulations currently recommended for use during pregnancy.

An additional benefit of maternal immunization is the transfer of protective antibodies through the placenta and breast milk, providing passive immunity during the first months of life. This protection is particularly important because infants younger than six months are not eligible for routine influenza vaccination and remain at increased risk of severe influenza-related complications. Collectively, current evidence supports maternal influenza vaccination as one of the most effective preventive strategies available to reduce the burden of influenza in both mothers and infants.

**Table 3:** Studies on influenza vaccination during pregnancy.

Study	Design	Sample	Outcomes Evaluated	Main Findings	Interpretation	Key Limitations
Omer et al., 2020 [11]	Pooled analysis of randomized clinical trials	10,002 pregnant women and 9800 liveborn infants	Maternal and neonatal protection	Vaccine effectiveness: 35–56% in infants and ~50% in mothers	Supports maternal immunization	Different epidemiological settings and influenza seasons.
Muthiani et al., 2023 [26]	Systematic review	Multiple RCTs	Perinatal outcomes	No significant reduction in low birth weight or prematurity	Vaccine mainly prevents infection rather than obstetric outcomes	Variation among included trials and outcomes assessed.
Motsoeneng et al., 2024 [27]	Clinical immunological study	283 pregnant women (138 vaccinated, 145 placebo)	Immune response after vaccination	Significant increase in antibody markers and lower infection risk	Demonstrates biological protection mechanisms	Immunological endpoints rather than clinical outcomes.
Chittajallu et al., 2025 [14]	Systematic review	8 studies	Immunogenicity and safety	Maternal seroprotection 65–95%; adverse effects < 10%	Vaccination considered safe and effective	Limited number of included studies and heterogeneity of vaccines evaluated.
Deese et al., 2025 [13]	Systematic review	18 studies	Breast milk antibodies	Increased anti-influenza IgA in breast milk	Supports passive neonatal immunity	Limited evidence directly linking breast milk antibodies to reduction in infant disease.

RCTs: randomized controlled trials.

### 3.4. Antiviral Treatment During Pregnancy

Regarding antiviral treatment (Table 4), Lian et al. (2022) [28] demonstrated that neuraminidase inhibitors, particularly oseltamivir, did not increase the risk of congenital malformations, low Apgar scores or prematurity, supporting their safety during pregnancy.

### 3.5. Biological Mechanisms Potentially Linking Influenza Infection and Adverse Outcomes

Several pathways have been proposed to explain the biological association between maternal influenza infection and adverse pregnancy outcomes (Table 5). Experimental and epidemiological studies suggest that maternal immune activation may lead to increased production of pro-inflammatory cytokines, including interleukin-6, tumor necrosis factor-α, and interferon-mediated responses, which may alter placental function and fetal development [9,20,29]. Maternal hyperthermia occurring during the first trimester has also been identified as a potential teratogenic factor associated with neural tube defects and other congenital anomalies [8,26]. Furthermore, oxidative stress, ferroptosis-related mechanisms, and placental vascular dysfunction may contribute to impaired fetal oxygenation and nutrient delivery, thereby increasing the risk of prematurity, fetal growth impairment, and congenital malformations [20].

Additionally, immune dysregulation during gestation has been hypothesized to alter fetal neurodevelopment, although evidence supporting long-term neuropsychiatric effects remains inconclusive.

Although these mechanisms are biologically plausible, their relative contribution remains incompletely understood and further mechanistic studies are warranted.

### 3.6. Public Health Implications and Limitations of Current Evidence

The findings included in this review reinforce the importance of early diagnosis, close obstetric monitoring and preventive vaccination strategies during pregnancy. The available evidence has several important implications for clinical practice and public health. First, pregnant women should continue to be considered a priority population for seasonal influenza prevention strategies because influenza infection is associated with increased risks of hospitalization, pregnancy complications, and adverse neonatal outcomes. Maternal influenza vaccination remains the most effective preventive intervention currently available and provides protection not only to pregnant women but also to infants during the first months of life through passive antibody transfer. In addition, healthcare provider recommendation and educational interventions remain essential to address vaccine hesitancy and improve vaccination uptake among pregnant women. Maternal vaccination should continue to be promoted as a priority public health intervention because it provides protection for both mothers and infants during the first months of life.

Early antiviral treatment with neuraminidase inhibitors, particularly oseltamivir, remains an important complementary strategy for the management of pregnant women with suspected or confirmed influenza infection. Current evidence suggests that timely treatment may reduce the risk of severe disease progression and hospitalization without increasing the risk of adverse fetal or neonatal outcomes. However, data regarding treatment effectiveness in pregnancy remain more limited than those available for vaccine effectiveness, highlighting the need for further research in this area.

Furthermore, increased obstetric surveillance may be warranted following maternal influenza infection during the first trimester because several studies have reported an association between early gestational influenza infection and an increased risk of congenital malformations. This recommendation relates to the timing of influenza infection rather than to antiviral treatment, as current evidence does not indicate an increased risk of congenital malformations associated with oseltamivir use during pregnancy.

Several limitations of the current evidence base should also be acknowledged. Considerable heterogeneity exists among studies regarding influenza diagnostic methods, study design, outcome definitions, and adjustment for confounding factors. Most available evidence derives from observational studies, limiting causal inference and increasing susceptibility to residual confounding factors such as maternal obesity, smoking status and socioeconomic conditions. Additional limitations include recall bias in studies relying on self-reported infection, limited long-term follow-up for neurodevelopmental outcomes, and potential publication bias. Furthermore, overlap among primary studies included in different systematic reviews and meta-analyses cannot be completely excluded. Future prospective studies using standardized methodologies and laboratory-confirmed influenza diagnoses are needed to strengthen the evidence base and improve comparability across studies.

## 4. Discussion

The present review summarizes current evidence regarding seasonal influenza infection during pregnancy and the role of maternal influenza vaccination as a preventive strategy. Overall, the available literature indicates that influenza infection during gestation is associated more consistently with adverse obstetric and congenital outcomes than with long-term neuropsychiatric disorders in offspring. At the same time, maternal vaccination demonstrates a favorable safety and effectiveness profile, reinforcing current international recommendations supporting immunization during pregnancy [11,12].

The findings of this review are broadly consistent with evidence published before 2020, which had already identified pregnant women as a high-risk population for influenza-related complications and supported maternal influenza vaccination as a safe preventive strategy. Earlier systematic reviews similarly reported associations between maternal influenza infection and adverse pregnancy outcomes, including prematurity and hospitalization. However, the more recent literature included in the present review provides additional evidence regarding congenital anomalies, neurodevelopmental outcomes, immunological mechanisms, and passive infant protection through maternal vaccination and breastfeeding. These newer studies also contribute to a better understanding of vaccine effectiveness and biological pathways potentially linking maternal infection and adverse fetal outcomes.

One of the most relevant findings of this review is the association between maternal influenza infection and adverse pregnancy outcomes, particularly spontaneous abortion and preterm birth. Wang et al. reported a significantly increased risk of miscarriage among infected pregnant women (OR 1.38), while Wang et al. identified a positive association between influenza infection and prematurity (OR 1.52) [6,7]. These findings are biologically plausible because maternal influenza infection may induce systemic inflammation, oxidative stress and placental dysfunction, which could trigger premature labor or compromise fetal viability [8]. Jiang et al. further supported these mechanisms by demonstrating the role of ferroptosis and inflammatory pathways in fetal developmental injury associated with H1N1 infection [20].

Pregnancy itself also appears to increase susceptibility to severe influenza infection. Gao et al. identified pregnancy as an independent risk factor for hospitalization among influenza patients [21]. This observation is consistent with the physiological and immunological adaptations of pregnancy, including reduced pulmonary reserve and altered immune responses, which may contribute to more severe respiratory disease [2,3]. Historical evidence from the 1918 and 2009 influenza pandemics similarly demonstrated increased morbidity and mortality among pregnant women [4,5].

Although the association between influenza infection and adverse obstetric outcomes appears relatively consistent across studies, the strength of these associations varies considerably. Differences in study design, influenza diagnostic criteria, population characteristics, vaccination coverage, and adjustment for confounding variables may partially explain this heterogeneity. Furthermore, many of the available studies are observational in nature, making it difficult to completely exclude residual confounding factors such as maternal age, obesity, smoking status, socioeconomic conditions, or access to healthcare services. Therefore, the observed associations should be interpreted cautiously and should not be considered definitive evidence of causality.

Regarding congenital malformations, the evidence included in this review was relatively consistent, particularly concerning first-trimester infection. Mátrai et al. [8] observed increased risks of neural tube defects, spina bifida, cleft lip/palate and congenital heart defects following maternal influenza infection during early pregnancy. Likewise, Su et al. confirmed a significant association between first-trimester influenza infection and congenital heart defects [23]. Arabzadeh et al. [24] also classified maternal influenza infection as a highly suggestive risk factor for neural tube defects.

These associations may be explained by several biological mechanisms. Current evidence suggests an association between first-trimester maternal influenza infection and an increased risk of congenital malformations, particularly neural tube defects and congenital heart defects. However, the available evidence is predominantly observational and does not establish causality. Maternal hyperthermia during organogenesis has long been considered teratogenic, while placental inflammation, oxidative stress and vascular injury may compromise fetal oxygen and nutrient supply [8,24]. The first trimester represents a particularly vulnerable developmental window because organogenesis and neural tube closure occur during this period.

However, it is important to recognize that most evidence linking maternal influenza infection with congenital malformations originating from observational studies and meta-analyses of observational data. Although biological plausibility exists, particularly through mechanisms involving maternal hyperthermia, inflammation, and placental dysfunction, the currently available evidence primarily supports an association rather than a direct causal relationship. Future prospective studies using laboratory-confirmed influenza diagnoses and rigorous adjustment for potential confounders are needed to better clarify these relationships.

When interpreting these findings, it is important to consider the relative strength of the available evidence. Associations between maternal influenza infection and congenital malformations are supported primarily by systematic reviews and meta-analyses of observational studies, which provide a higher level of evidence than individual studies but remain susceptible to residual confounding and exposure misclassification. In contrast, evidence regarding neurodevelopmental outcomes is considerably less consistent. Variability in study design, follow-up duration, diagnostic criteria, and methods used to ascertain maternal influenza infection likely contributes to the heterogeneous findings reported across studies. Consequently, the evidence supporting congenital anomalies appears more robust than that relating to long-term neurodevelopmental disorders.

In contrast, evidence linking maternal influenza infection with long-term neurodevelopmental and neuropsychiatric disorders remains inconsistent. Fung et al. found inconclusive association between prenatal influenza exposure and autism spectrum disorder, attention-deficit/hyperactivity disorder or schizophrenia [10]. Similarly, San Martín-González et al. [9] reported heterogeneous findings regarding psychomotor and neurodevelopmental outcomes. Although maternal immune activation and cytokine-mediated fetal brain injury have been proposed as possible mechanisms [29], current epidemiological evidence does not support definitive causal conclusions.

Several important uncertainties remain. First, many studies rely on self-reported influenza infection or clinical diagnosis rather than laboratory-confirmed infection, increasing the possibility of exposure misclassification. Second, long-term neurodevelopmental outcomes require prolonged follow-up periods, which are not available in many cohorts. Third, residual confounding factors, including maternal comorbidities, socioeconomic status, smoking, obesity, and concurrent infections, may influence observed associations. These limitations should be considered when interpreting the current evidence and highlight the need for well-designed prospective studies with standardized outcome definitions and laboratory-confirmed exposure assessment.

These inconsistencies may reflect methodological heterogeneity, variable exposure definitions and limited long-term follow-up across studies. An additional challenge in interpreting neurodevelopmental outcomes is the long latency period required before many neurological and psychiatric conditions can be reliably diagnosed. Consequently, many studies may not have sufficient follow-up duration to detect subtle developmental effects. Variability in outcome assessment methods, diagnostic criteria, and population characteristics may further contribute to the inconsistent findings reported in the literature. At present, the available evidence remains insufficient to establish a definitive relationship between prenatal influenza exposure and long-term neurodevelopmental disorders.

Another important finding concerns antiviral treatment during pregnancy. Lian et al. [28] demonstrated that neuraminidase inhibitors, especially oseltamivir, did not increase risks of congenital malformations, prematurity or adverse neonatal outcomes. These findings support current clinical guidelines recommending early antiviral treatment in pregnant women with suspected or confirmed influenza infection, particularly in severe cases or high-risk patients.

Maternal influenza vaccination emerged as one of the strongest and most consistent protective interventions identified in this review. Beyond the evidence summarized in individual studies, ongoing influenza vaccine research continues to focus on improving vaccine effectiveness across seasons. One of the major challenges in influenza prevention is the continuous evolution of circulating influenza viruses through antigenic drift, which may reduce the match between vaccine strains and circulating viruses. Less frequently, antigenic shift may generate novel influenza strains with pandemic potential. Consequently, seasonal influenza vaccines require annual reformulation based on global surveillance data and predictions regarding predominant circulating strains. Although vaccine effectiveness may vary between seasons, maternal influenza vaccination consistently provides clinically meaningful protection against influenza infection, severe disease, and influenza-related hospitalization in both mothers and infants.

Recent research has also explored the development of broader and potentially universal influenza vaccines capable of providing protection against multiple influenza strains. While these approaches remain under investigation, current seasonal influenza vaccines continue to represent the most effective preventive strategy available for pregnant women. Therefore, maintaining high vaccination coverage remains a priority public health objective.

Omer et al. [11] demonstrated vaccine effectiveness ranging from 35% to 56% in infants during the first six months of life and approximately 50% effectiveness in mothers. This neonatal protection is especially relevant because infants younger than six months are not eligible for influenza vaccination and remain highly vulnerable to severe influenza complications.

In addition to clinical effectiveness, vaccination demonstrated a favorable safety profile. Chittajallu et al. [14] reported maternal seroprotection rates between 65% and 95%, while adverse effects remained generally mild and occurred in less than 10% of vaccinated women. Importantly, vaccination was not associated with increased risks of prematurity, low birth weight or fetal death [11,26]. These findings strongly reinforce current recommendations from the WHO and CDC advocating influenza vaccination during any trimester of pregnancy [1,12].

The immunological mechanisms underlying maternal vaccination are also clinically relevant. Motsoeneng et al. [27] demonstrated that vaccination induced measurable increases in antibody-related immune markers associated with reduced influenza infection risk. Furthermore, Deese et al. [13] observed increased anti-influenza IgA antibodies in breast milk following maternal vaccination, supporting an additional mechanism of passive neonatal protection during breastfeeding.

Despite the substantial evidence supporting vaccine safety and effectiveness, vaccination coverage among pregnant women remains suboptimal in many settings. Vaccine hesitancy continues to be influenced by concerns regarding fetal safety, misinformation, and insufficient healthcare provider recommendations. Several studies have demonstrated that healthcare professional endorsement remains one of the strongest predictors of maternal vaccine acceptance. Therefore, educational interventions targeting both pregnant women and healthcare providers remain essential components of maternal immunization programs.

Despite these important findings, several limitations should be acknowledged. First, considerable heterogeneity exists among studies regarding influenza diagnostic criteria, study design and outcome definitions, making direct comparisons difficult. Second, most available evidence derives from observational studies, limiting causal inference and increasing susceptibility to confounding factors such as maternal obesity, smoking status and socioeconomic conditions. Third, recall bias may affect studies based on maternal self-reported influenza infection. Another important consideration is that a substantial proportion of the literature included in this review was published during the COVID-19 pandemic period. Public health interventions implemented to control SARS-CoV-2 transmission, including social distancing measures, mask use, travel restrictions, and enhanced hygiene practices, dramatically altered influenza circulation worldwide. Furthermore, SARS-CoV-2 infection itself has been associated with adverse pregnancy outcomes, including prematurity and maternal morbidity. Consequently, distinguishing the specific contribution of influenza infection from that of SARS-CoV-2 may have been challenging in some studies conducted during this period, potentially introducing additional sources of bias and confounding. Finally, evidence regarding long-term neurodevelopmental outcomes remains limited because of insufficient longitudinal follow-up and methodological variability.

Because this study was conducted as a narrative review, the methodology was not intended to meet all requirements of a formal systematic review, and therefore the findings should be interpreted within this context. An additional limitation should be acknowledged. Several of the studies included in this review were systematic reviews, umbrella reviews, and meta-analyses that may have incorporated some of the same primary studies. Consequently, partial overlap of the underlying evidence base cannot be completely excluded. Although the objective of the present narrative review was to summarize the current body of evidence rather than to quantitatively pool results, this potential overlap may have increased the prominence of certain findings reported consistently across multiple reviews.

However, this review has several strengths. It synthesizes recent evidence published between 2020 and 2025, incorporates high-quality systematic reviews and meta-analyses, and integrates clinical findings with biologically plausible mechanisms. Additionally, the structured thematic organization facilitates interpretation of maternal, fetal and vaccination-related outcomes. Overall, the available evidence is strongest for the effectiveness and safety of maternal influenza vaccination and for the association between maternal influenza infection and adverse obstetric outcomes, including prematurity and hospitalization. Greater uncertainty remains regarding congenital malformations and especially long-term neurodevelopmental consequences, where methodological heterogeneity and limitations of the available studies preclude definitive conclusions. Future research should focus not only on confirming these associations but also on clarifying the biological mechanisms underlying maternal-fetal interactions during influenza infection.

From a public health perspective, the evidence summarized in this review supports continued promotion of maternal influenza vaccination as a safe and effective preventive strategy. Educational interventions addressing vaccine hesitancy among pregnant women and healthcare professionals remain essential to improve vaccination coverage and reduce influenza-related maternal and neonatal morbidity. Another limitation of the current evidence base is the potential overlap of primary studies included within different systematic reviews and meta-analyses. Because several reviews evaluate similar populations and outcomes, some degree of duplication cannot be excluded when synthesizing findings across studies. This issue should be considered when interpreting the overall weight of the evidence presented in this review.

Future research should prioritize large prospective longitudinal studies using standardized methodologies and laboratory-confirmed influenza diagnosis. Further investigation is also needed regarding the long-term neurodevelopmental effects of prenatal influenza exposure and the immunological mechanisms underlying maternal-fetal protection induced by vaccination.

The main contribution of the present review lies in its synthesis of evidence published between 2020 and 2025, a period characterized by substantial advances in maternal immunization research and by the influence of the COVID-19 pandemic on infectious disease epidemiology. By integrating data from systematic reviews, meta-analyses, randomized clinical trials, and mechanistic studies, this review provides an updated overview of both the risks associated with influenza infection during pregnancy and the benefits of maternal vaccination. The findings reinforce previous recommendations while highlighting areas where uncertainty persists and additional research remains necessary.

## 5. Conclusions

Seasonal influenza infection during pregnancy remains an important clinical and public health concern.

Current evidence supports an association between maternal influenza infection and adverse obstetric outcomes, including spontaneous abortion, prematurity and hospitalization. First-trimester infection may increase risks of congenital malformations, particularly neural tube defects and congenital heart defects.

Evidence regarding long-term neurodevelopmental consequences in offspring remains inconclusive.

Maternal influenza vaccination demonstrates a favorable safety profile and provides effective protection for both mothers and infants. In addition to reducing maternal influenza infection risk, vaccination contributes to passive neonatal immunity through placental and breast milk antibody transfer.

These findings reinforce current recommendations supporting universal influenza vaccination during pregnancy.

## Figures and Tables

**Table 4:** Studies on antiviral treatment during pregnancy.

Study	Design	Sample	Outcomes Evaluated	Main Findings	Interpretation	Key Limitations
Lian et al., 2022 [28]	Meta-analysis	9 studies; Pregnant women treated with antivirals	Neonatal safety	No increased risk of congenital malformations or low Apgar scores	Supports safety of oseltamivir during pregnancy	Based on observational cohort studies vulnerable to confounding by indication and other biases.

**Table 5:** Biological mechanisms potentially linking influenza infection during pregnancy and adverse outcomes.

Mechanism	Proposed Effect	Potential Consequences
Maternal systemic inflammation	Increased cytokine production	Placental dysfunction and fetal inflammatory exposure
Maternal hyperthermia	Teratogenic effect during organogenesis	Neural tube defects and congenital malformations
Oxidative stress	Cellular and placental damage	Fetal growth impairment and prematurity
Placental vascular injury	Reduced oxygen and nutrient supply	Congenital heart defects and fetal hypoxia
Immune dysregulation	Altered fetal neurodevelopment	Possible neuropsychiatric and developmental effects

## Data Availability

No new data were created or analyzed in this study.
